# Effects of Age on the Detection and Management of Breast Cancer

**DOI:** 10.3390/cancers7020815

**Published:** 2015-05-22

**Authors:** Andrew McGuire, James A. L. Brown, Carmel Malone, Ray McLaughlin, Michael J. Kerin

**Affiliations:** Discipline of Surgery, School of Medicine, National University of Ireland, Galway, Ireland; E-Mails: andrewmcguire@rcsi.ie (A.M.); carmel.malone@nuigalway.ie (C.M.); ray.mclaughlin@hse.ie (R.M.)

**Keywords:** breast cancer, age, subtype, screening, miRNA

## Abstract

Currently, breast cancer affects approximately 12% of women worldwide. While the incidence of breast cancer rises with age, a younger age at diagnosis is linked to increased mortality. We discuss age related factors affecting breast cancer diagnosis, management and treatment, exploring key concepts and identifying critical areas requiring further research. We examine age as a factor in breast cancer diagnosis and treatment relating it to factors such as genetic status, breast cancer subtype, hormone factors and nodal status. We examine the effects of age as seen through the adoption of population wide breast cancer screening programs. Assessing the incidence rates of each breast cancer subtype, in the context of age, we examine the observed correlations. We explore how age affects patient’s prognosis, exploring the effects of age on stage and subtype incidence. Finally we discuss the future of breast cancer diagnosis and treatment, examining the potential of emerging tests and technologies (such as microRNA) and how novel research findings are being translated into clinically relevant practices.

## 1. Introduction

Breast cancer is the most commonly diagnosed cancer and the leading cause of cancer deaths in women worldwide, accounting for 23% of total cancer cases and 14% of all cancer related mortalities [1]. Currently, the lifetime risk of developing breast cancer for women is 1/8. However, >40% of the affected patients are currently >65 of age and remarkably, this group accounts for almost 60% of the total deaths from breast cancer [2,3]. Interestingly, before 49 years of age the estimated risk of developing breast cancer is 1/53 however, this rises to 1/43 for 50–59 years old and rises again to 1/23 for 60–69. Significantly, for women aged >70 this risk is the highest with a 1/15 chance of developing breast cancer [3].

The number of elderly patients with breast cancer is due to rapidly increase in the near future, as more than 20% of the population expected to be >65 years old by 2030 [4]. Furthermore, improvements in disease screening and diagnosis mean increasing numbers of the population have breast cancer detected and at a steadily earlier age. Together, these trends are resulting in a greater number of, often elderly, patients requiring long-term treatment or management of breast cancer. In this review we will examine the effects of modern investigations and treatment options for breast cancer, categorised by age group and how this may influence changes in future research and treatment options.

## 2. Population Based Screening and Age 

Currently breast cancer screening programs are running in >26 countries across the world (Table 1: 25 countries shown; modified from [5,6] though debate remains over the efficacy of some of these programs, what sections of the population should be screened and at what age the screening should be performed [6,7,8,9,10]). The introduction of early detection breast cancer screening programs has resulted in increased breast cancer detection rates for all age groups. Numerous studies investigating the benefits of screening programs have demonstrated a reduction in mortality rates, with maximal benefit seen in women aged 50–70 years [8,11,12].

Based on current evidence, full field digital mammography (FFDM) is the gold standard for breast cancer screening [13]. Current clinical recommendations from the U.S. Preventive Services Task Force are: biennial screening for all women aged 50–75 years old [14]. Some debate remains as to whether screening should continue past 70 years of age. However, comparison of screened *versus* non-screened breast cancer patients >70 years old shows a significant advantage for the screened cohort, with breast cancer diagnosed at an earlier stage, leading to a considerably reduced mortality rate [15,16]. However, it has been suggested that the reduced mortality could be due to improved adjuvant treatment [17,18,19]. Presently, there is insufficient data available to make a formal recommendation [20,21]. Due to the benefits observed from screening programs many countries have increased the age range of patients screened, with the United Kingdom due to extend its program to cover women aged 45–73 (by 2016). However, the particulars of screening remains controversial, with the best results observed using double reading and two projections [9,22]. While mammography has an overall sensitivity of ~79%, this is reduced in younger women and women with dense breast tissue [23,24,25] (Table 2). Newer imaging techniques have emerged over the last few years such as tomosynthesis, contrast enhanced spectrum mammography and automated whole breast ultra-sound [26,27,28]. Yet, there is still insufficient data on these new techniques to change current practices.

**Table 1:** Countries with breast cancer screening programs.

Country	Screening introduced	Ages Screened	Interval (Yrs)	Population screened (annually)
Australia	1991	40–75+	2	1,700,000 *
Canada	1988	50–69	2	196,187
China	2009	40–59	3	1,200,00
Denmark	1991	50–69	2	275,000
Finland	1987	60–64	2	N/A
France	1989	50–74	2	2,343,980
Iceland	1987	40–69	2	20,517
Israel	1997	50–74	2	220,000
Italy	2002	50–69	2	1,340,311
Japan	1977	40–75+	2	2,492,868
Korea	1999	40–75+	2	2,602,928
Luxembourg	1992	50–69	2	14,586
Netherlands	1989	50–74	2	961,786
New Zealand	1998	45–69	2	211,922
Norway	1996	50–69	2	199,818
Poland	2006	50–69	2	985,364
Portugal	1990	45–69	2	100,364
Rep of Ireland	2000	50–64	2	28,794
Saudi Arabia	2007	40–64	2	6200
Spain	1990	45–69	2	527,000
Sweden	1986	40–74	2	1,414,000
Switzerland	1999	50–69	2	60,700
United Kingdom	1988	50–69	3	1,957,124
United States of America	1995	40–75+	1–2	416,000
Uruguay	1990	40–69	1	352,000

* 50–69 year olds.

**Table 2:** Breast cancer screening programs and detection rates.

Age Group	Digital Mammography		Magnetic Resonance Imaging	
	Sensitivities [23,24,29]	Clinical Guidelines	Sensitivities [7,30,31]	Clinical Guidelines
<40	54%–77%	Familiar history of breast cancer	71.1%–77.3%	Familiar history of breast cancer
40–49	77%–86%			
50–59	78%–93%	Biennial screening (between the ages of 50–75)		Biennial screening (between the ages of 50–75)
60–69	78%–94%			
>70	81%–91%			

As the sensitivity of mammography is reduced in younger women [23], it is currently recommended that high-risk patients have yearly MRI and mammograms (alternating every 6 months), which has been shown to increase detection rates [30,31,32,33]. Specifically related to age, a 77% sensitivity was observed in 35–55 year old women [30]. However overall, the sensitivity of MRI ranges between 71%–77.3% in breast cancer detection, although this can be increased to 94% when combined with mammography [7,30,31]. Based on the figures presented (Table 2) digital mammography is the superior method for detecting breast cancer, except in young patients where there is a high degree of variability. This is reflected in clinical practice where digital mammography is the most commonly used diagnostic tool for older patients (>50 years old).

**Figure 1:**
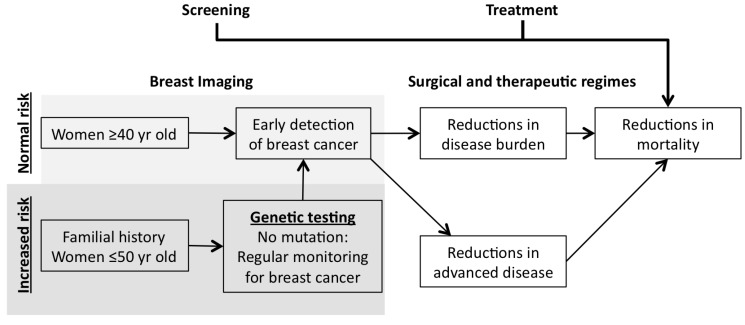
Overview of current breast cancer screening practices.

Screening of high-risk patients with MRI does have drawbacks, as MRI is time consuming, expensive (compared to mammography alone) and has a lower specificity (resulting in a large number of benign biopsies) [30,31,34,35]. While screening may detect cancers in high-risk age groups, patients with a family history of breast or ovarian cancer are at a higher-risk than the general population. Augmenting the physical screening programs are the recent advances in genetic analysis. Over the last decade the discovery of genetic testing for breast cancer susceptibility genes (such as *BRCA1* & *BRCA2*) has seen a rise in preemptive screening in many countries, particularly where a strong family history of breast cancer has been observed [36,37] (Figure 1).

A significant amount of research conducted in the last 20–30 years has been dedicated to expanding our knowledge of the underlying molecular mechanisms and genetic risk factors influencing breast cancer susceptibility and development [38,39]. Significantly however, very little research has focused on the effects of age on these molecular mechanisms. In this context this research is further complicated by the additional factors such as reproductive status, menarche and menopause, which can be difficult to mimic in a research setting [40].

Moreover, many clinical trials have evaluated new diagnostic tests and treatment options for breast cancer. However, many randomized clinical trials investigating breast cancer used patients from younger age groups [41,42,43]. While management of younger patients has been greatly investigated, the treatment options for older patients remain largely a clinical-based decision. Often this is related to the stage of disease and the patient’s general health [44,45,46]. Clearly some key questions which remain to be answered are: Do treatment options affect breast cancer patients survival based on age group? And is breast cancer subtype affected by age?

## 3. Incidence and Lifetime Risk of Breast Cancer by Molecular Subtype and Age

Molecular profiling has resulted in breast cancer being divided into four main subtypes, defined by differing expression levels of the Estrogen receptor (ER), Progesterone receptor (PR) and growth factor receptor HER2. The subtypes are: Luminal A (ER+ve/PR+ve/HER2-ve), Luminal B (ER+ve/PR+ve/ HER2+ve), HER2 over-expressing (ER-ve/PR-ve/HER2+ve) and basal (triple negative: ER-ve/PR-ve/ HER2-ve) [47,48]. Luminal cancers are most common breast cancer seen (70%–80%) followed by HER2 over-expressing (10%–20%) and approximately 10% are basal cancers [49,50]. Currently, the incidence of each molecular subtype has been demonstrated to vary by age group (summarized in Table 3, [51]). Recently, molecular testing of breast cancer has further confirmed these trends [50].

**Table 3:** Breast cancer incidence and lifetime risk by molecular subtype and age.

Breast cancer molecular subtype	Breast cancer incidence by age group					Lifetime risk (by subtype)
	<40	40–49	50–59	60–69	>70	
Luminal A	2.9%	14.2%	28.3%	**31.9%**	**22.7%**	6.79% (Luminal A & B)
Luminal B	8.1%	20.7%	32.4%	20.8%	17.9%	
HER2	5.5%	16.3%	31.6%	28.8%	17.8%	1.78%
Triple Negative	**10.8%**	**26.5%**	**35.0%**	17.5%	10.1%	1.2%

Perhaps unsurprisingly, the under 40 age group is most likely to present with the more aggressive resistant triple negative breast cancer subtype (10.8% incidence rate, at an almost ~2 fold higher risk of the next most common subtype Her2) (Table 3, bold text). Interestingly, this trend continues until the age of 60, however from the age of 60 onwards Luminal A has the highest incidence rate (Table 3, bold text). Surprisingly, for the under 50’s Luminal A is the least common breast cancer subtype (Table 2), although this is strongly influenced by the very small numbers of breast cancers seen in these age brackets (~7% to the total cases) [52,53]. In the 50–59 age bracket Triple negative is again the most common subtype observed (35.0%), while Luminal A is the least common subtype (28.3%), however there is an almost equal chance of developing any of the subtypes. Currently, in the 60–69 and >70’s age groups the breast cancer subtypes with highest incidence is Luminal A (31.9% and 22.7%) with Triple negative now the least common subtype observed (17.5% and 10.1%). As expected, the most common form of breast cancer observed is the Luminal subtype (6.79%) with triple negative the least common (1.2%) (Far right column) [54].

Notably, breast cancer survival is strongly associated with age at diagnosis (Table 4) [55]. Lower survival is seen in patients under 50, while patients over 70 have the lowest survival. The poor survival in the >70’s group is certainly influenced by their age and their age related co-morbidities (discussed below in Section 7).

Investigating how high risk genetic mutations effect the age of onset we find that in patients <40 years old 5.3% of breast cancer cases are due to mutations in the BReast CAncer susceptibility gene 1 (*BRCA1*). In the 40–49 age bracket this falls to 2.2%, which decreases further to 1.1% for patients developing breast cancer in the 50–70 year age group [56]. Furthermore, it has been established that patients with *BRCA1* mutations are more likely to develop basal like breast cancers (including the triple negative molecular subtype) [57,58,59].

**Table 4:** Five Year Survival rates for breast cancer by age [55].

Age Group	5 Year survival (%)
<40	84.5
40–49	89.4
50–59	90.9
60–69	90.8
>70	73

## 4. Genetics and Breast Cancer Risk

**Figure 2:**
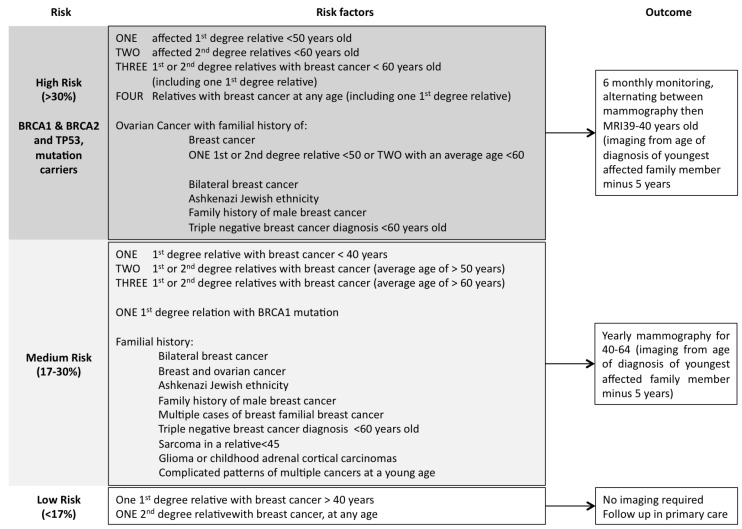
Family history, breast cancer risk and screening.

Currently the major genes known to influence breast cancer risk is *BRCA1* [60] and *BRCA2* [61,62]. These genes are tumour suppressor genes responsible for DNA damage repair [63] and mutations in these genes result in a significantly increased risk of breast cancer. It is estimated that up 16% of all familial breast cancers are due to mutations in these genes [64] and up to 5% of all breast cancer cases [65]. *BRCA1* and *BRCA2* mutation carriers <70 years old face a 57% and 49% (respectively) risk of developing breast cancer [66]. Importantly, *BRCA* mutation carriers frequently tend to develop a more aggressive breast cancer and at a younger age [67]. Screening for *BRCA* gene mutations in high-risk patients has become a priority and scoring systems such as the Manchester scoring system provides a means to identify which patients need increased surveillance [68]. From scoring systems like this, Genetic testing guidelines have recently been introduced for higher-risk patients (Figure 2).

Current recommendations for patients with detected *BRCA* mutations are bilateral mastectomies for carriers [69,70,71], with patients who decline surgery to continue high-risk screening. Additionally, genetic screening for first degree relatives is recommended [72]. The genetic testing of patients can have significant personal ramifications, in addition to the consequences for their families and close relatives. Due to this genetic screening is not routine worldwide, with genetic counseling recommended prior to testing [71].

## 5. Breast Cancer and microRNAs

MicroRNA (miRNA) are 19–25 long non-protein coding RNA involved in cell development, differentiation, proliferation and apoptosis [73,74,75]. Currently >2000 distinct miRNAs have been identified in humans, where miRNAs regulate an estimated 30% of all human genes [76]. Recently age has been implicated as a factor affecting the differential expression of miRNAs [77,78,79]. Current research has focused on the role of miRNAs and breast cancer, implicating aberrant miRNA regulation as a factor in breast cancer initiation and progression [80,81,82,83]. Recently, a single study has investigated variations in circulating miRNA (in the blood), finding distinct differences in the circulating miRNA profiles of younger and older breast cancer patients [84]. Currently, miRNAs are being explored as new potential therapeutic targets or treatment options for breast cancer. Further research into the effects of age, circulating miRNA and breast cancer may further additional insight into the variations, diagnosis or treatment options for breast cancer patients of distinct age groups. This research represents another valuable step towards personalized treatment options.

As breast cancer is a heterogeneous disease, genetic insights have complemented the research focused on sub-classifying breast cancer by molecular subtype. Molecular profiling has identified new breast cancer subtypes and has the potential to explain the difference seen in subtypes across the age groups.

## 6. Age Associated Treatment by Molecular Subtype

Luminal cancers (A & B) are the most common subtypes and tend to occur in post-menopausal patients [50,51,85,86,87] and tend to have better outcomes than the other subtypes [51,85,88,89]. Furthermore, Luminal cancers have been linked to estrogen exposure, with nulliparous and women taking hormone replacement therapy displaying an increased risk [90,91]. Due to this anti-estrogen agents (such as tamoxifen) have been developed that inhibit estrogen activity by competitively binds to oestrogen receptors. This treatment has increased disease free survival and overall survival in hormone receptor positive cancers, with 5 years of adjuvant tamoxifen treatment reducing annual mortality by up to 31% across all age groups [92,93]. Interestingly, further studies have indicated that extended treatment with tamoxifen can have other age related effects, such as prevention of bone loss in post-menopausal women [92] and increasing the risk of endometrial cancers, hot flushes and thromboembolic events [92,94,95]. New anti-estrogen therapies (aromatase inhibitors) were developed which inhibit the synthesis of estrogen from androgens [96,97]. Long-term studies showed that aromatase inhibitors (such as letrozole) have superior disease free survival rates compared to tamoxifen, in post-menopausal women [98]. Letrozole also reduces the risk of endometrial cancer along with vaginal bleeding, cerebrovascular events, thromboembolic events and flushes [99,100]. Currently an extended course of hormone therapy, beyond five years, is recommended due to improved survival [101,102,103]. Previous studies have indicated that luminal cancers have a reduced sensitivity to chemotherapy [104]. The improved outcomes using tamoxifen and aromatase inhibitors pose a question about the use of chemotherapy for luminal cancers. The results from new tests, such as the multi-gene Oncotype Dx test, provides an estimate for the risk of cancer recurrence for an estrogen positive breast cancer patient. Furthermore this test also identifies patients that are either likely, or unlikely, to benefit from chemotherapy [105,106,107]. Use of this test in advanced age groups has resulted in a large proportion of elderly patients not receiving chemotherapy and improvements in quality of life.

HER2 over-expressing cancers have a higher prevalence in post-menopausal women (Table 3) and initially had poor outcomes, prior to the development of targeted treatments [51,108]. In patients under 40 years old, HER2 over-expressing breast cancers have been linked to a higher recurrence rate [109]. Treating patients with a monoclonal antibody that targets the Her2 receptor, like trastuzumab, has resulted in improved survival [110,111,112]. Furthermore, adding trastuzumab to neo-adjuvant chemotherapy has lead to significant increases in the pathological complete responses observed [113]. Development of newer monoclonal antibody treatments has also shown promise, as pertuzumab combined with trastuzumab in the neo-adjuvant setting significantly improves the pathological complete response rate [114].

Triple negative cancers occur at a younger age and have poorer outcomes than luminal subtypes [50,51,86,87]. Triple negative cancers have been linked to the *BRCA1* gene, with studies finding 20%–30% of triple negative patients having either the *BRCA1* or *BRCA2* gene [59,115,116]. It was also found that the prevalence increases with decreasing age [115,117]. Due to this correlation the national comprehensive cancer network recommends that all women under 60 with triple negative breast cancer be referred for genetic counseling [118]. Currently, bilateral prophylactic mastectomy has been shown to reduce the risk of breast cancer in carriers with *BRCA1* and *BRCA2* mutations [119].

**Figure 3:**
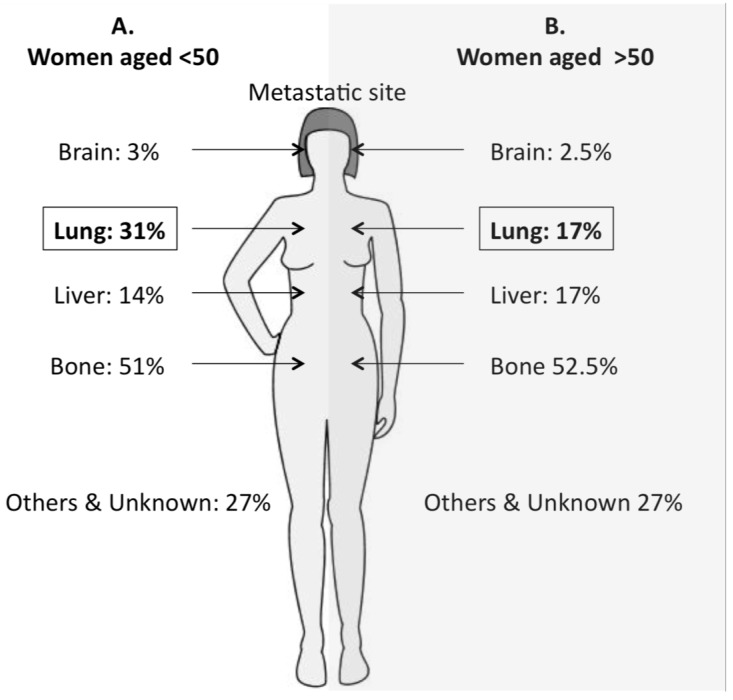
Metastatic breast cancer sites by age group.

A variation in rates of metastasis is seen across age groups, with older patients more likely to have distal metastasis [120]. However, by age (over or under 50 years old) there is little difference in the sites metastasis except for lung metastasis which is almost twice as more common in younger patients (Figure 3).

The most common surgical intervention for breast cancer treatment is wide local excision (WLE), which has similar outcomes to mastectomy while reducing surgical complications [121,122]. While no difference in survival is seen in premenopausal women having WLE, surprisingly there is a higher local recurrence rates with up to five fold greater incidence seen in women <35 compared to women aged 45–49 [123,124,125,126,127]. This can make treatment decisions difficult as younger patient would prefer to have breast conserving surgery. The margin status in breast conserving surgery has been shown to be one of the most significant factors in relapse rates. Clear surgical margins have been shown to dramatically reduce recurrence rates especially in women under 40 [127,128]. The addition of radiotherapy post WLE has reduced recurrence for women <50, falling significantly from 19.4% to 11.4% [129]. Interestingly, in patients >70 radiotherapy has not been shown to improve survival [130]. It has been shown that there is an age dependent response to chemotherapy and hormone therapy, where anthracycline-based polychemotherapy reduced mortality by 38% for women <50 years old and by 20% for the 51–69 years old group [131]. A similar improvement in survival rates was seen across all groups in ER positive breast cancers treated with tamoxifen [131]. In HER2 positive patients treated with trastuzumab, no significant difference is seen in recurrence rates across different age groups [132]. Neo-adjuvant chemotherapy reduces tumour size and increases the numbers of patients suitable for surgery, however no difference is seen by age for complete pathological response [133]. Neo-adjuvant endocrine therapy is of benefit in post-menopausal patients with early breast cancer, improving WLE rates, disease free survival and overall survival [134,135].

## 7. Prognosis

Prognosis of patients varies with age, as younger women tend to have more aggressive tumours (such as triple negative) and a higher recurrence rate [136,137,138,139,140,141]. This effect is most pronounced in women <35 years old and is has been demonstrated that a younger the age of diagnosis increases the risk of mortality [142]. These effects are likely due in part to the lack of screening for younger women, meaning patients often present with larger palpable lumps and a more advanced stage [143]. Younger patients tend to have higher Ki-67 levels (an indicator of poor prognostic outcome [144]), with highest levels seen in patients <35 [145,146]. However, contradicting this recent studies have shown no age related difference in mortality rates [147,148,149]. Nevertheless, current advances in screening, earlier identification of high-risk patients and improved treatment options may explain this. In addition, recent studies have shown that women >55 years old have a better prognosis and have a similar survival to the general population irrespective of disease status [150]. Mirroring the younger patients, women at the other end of the age spectrum >70 years old similarly present with more advanced tumours [46]. In younger patients chemotherapy would be used, however there is little research into which subgroups in this >70 age group this would be a suitable option for.

## 8. Age and co-Morbidities

In the past many older patients were deemed unsuitable for surgery due to their age and medical co-morbidities such as diabetes mellitus, coronary heart disease, hypertension, stroke, asthma and chronic gastritis. These co-morbidities are independent risk factors for survival and are disproportionally found increased in older patients [45,151,152]. Improved surgical techniques mean a larger proportion of these patients are now able to undergo curative surgery. These medical co-morbidities may provide markers for assessing suitability for chemotherapy, in conjunction with other established factors such as a comprehensive geriatric assessment (CGA). A recent study found malnutrition and frailty to be the biggest risk factor for mortality in patients >70 years old [153]. Similar results were seen in a study using a CGA in patients >65 years old where conditions such as a low Mini Mental State Examination, Body Mass Index or high Charlson co-morbidity index scores resulted in a higher risk of chemotherapy related toxicity [154]. A CGA may provide relevant age related information indicating which patients would be suitable for chemotherapy treatment. However, limitations may include not completing a CGA prior to treatment and non-compliance with recommendation [46].

## 9. Summary

With our ever-expanding knowledge of breast cancer and age related effects, there are constant improvements in treatment guidelines and best practice. Over the last few decades, detection and survival rates have improved immensely, yet there is no consensus in the management of the very young (<35) or the increasingly elderly (>70) populations. An improved understanding of the genetics of breast cancers through molecular profiling may provide information that can be applied to the youngest and oldest patients. This has been demonstrated by identification of the high-risk *BRCA* genes, providing some explanation for younger patients. Importantly, there are still no clear guidelines for the management of breast cancer patients >65 years old. In addition, scoring systems such as CGA or miRNA profiles could provide an accurate way of determining which patients should receive active or palliative treatment. Further investigations are needed to determine the feasibility and practicality of such systems, which are further steps towards truly individualized treatment plans.
